# Supplementary material for: Stability of Quantum-Dot Light Emitting Diodes with Alkali Metal Carbonates Blending in Mg Doped ZnO Electron Transport Layer
Source: Nanomaterials (Basel). 2020 Dec 4;10(12):2423. doi: 10.3390/nano10122423 (PMC7761844; doi:10.3390/nano10122423)
Supplement: Supplementary file 1 [file nanomaterials-10-02423-s001.pdf]

Supplementary Materials

The Stability of Quantum-dot Light Emitting Diodes with Alkali Metal Carbonates Blending in Mg doped ZnO Electron Transport Layer

Hyo-Min Kim, Wonkyeong Jeong, Joo Hyun Kim and Jin Jang

Department of Information Display and Advanced Display Research Center, Kyung Hee University, 26 Kyungheedae-ro, Dongdaemun-gu, Seoul, 02447, Korea

Keywords: Alkali Metal Carbonate, Metal oxide, Mg doped ZnO, Quantum-dot, QLED

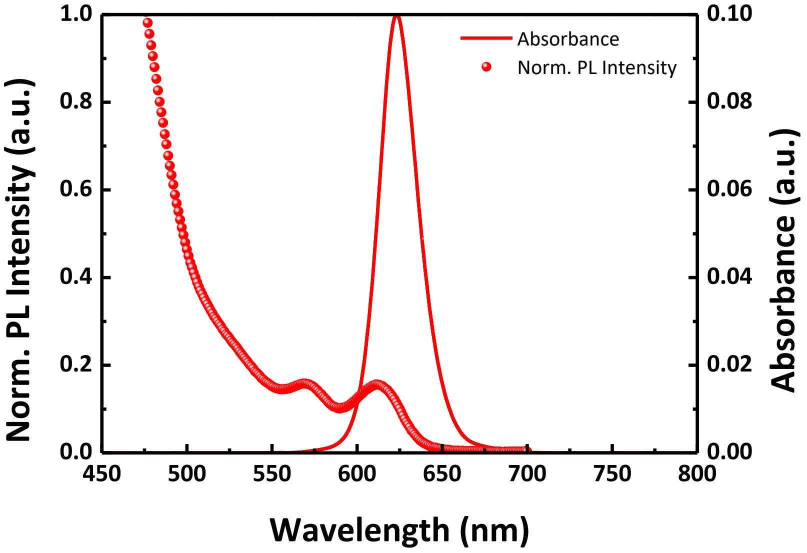

Figure S1. Absorbance and normalized PL characteristics of R-QDs used in this study.

Table S1. Chemical structure, molecular weight, melting point and boiling point characteristics of alkali metal carbonates used in this study.

**Alkali Metal Carbonate**	**Cs_2CO_3**	**Rb_2CO_3**	**K_2CO_3**	**Na_2CO_3**	**Li_2CO_3**
**Chemical structure**	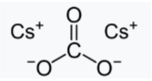	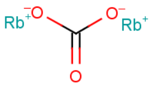	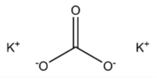	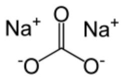	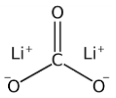
**M_w (g/mol)[1)]**	325.82	230.945	138.2	105.99	73.89
**m.p (°C)[2)]**	610	837	891	851	723
**b.p (°C)[3)]**	N/A	900	N/A	N/A	1310

[1)] M_w: Molecular weight
[2)] m.p: Melting point
[3)] b.p: Boiling point

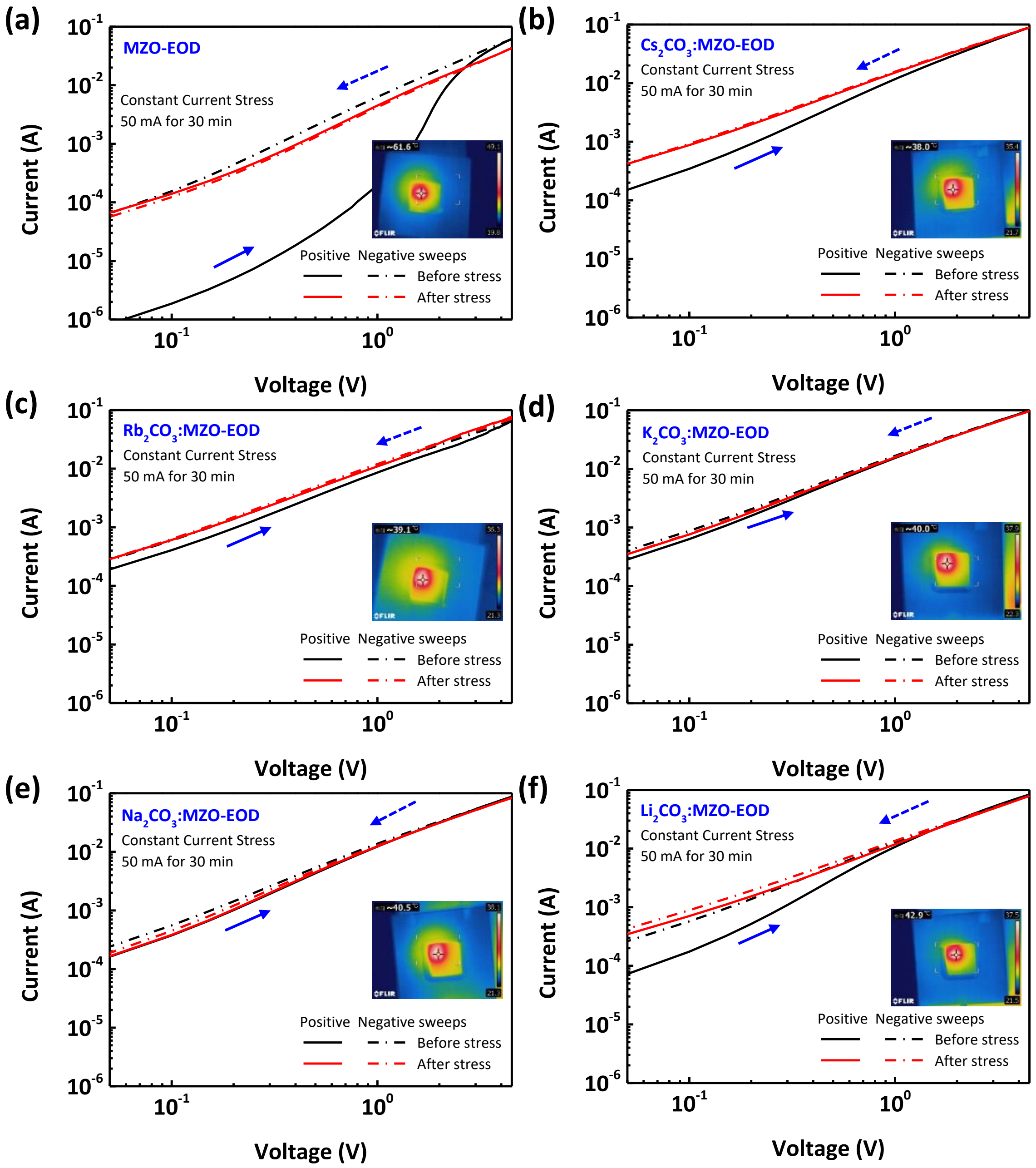

Figure S2. Electrical stability characteristics of EODs with alkali metal carbonate blended MZO ETL and QD EML. Current-voltage characteristic of EODs with (a) MZO, (b) Cs_2CO_3:MZO, (c) Rb_2CO_3:MZO, (d) K_2CO_3:MZO, (e) Na_2CO_3:MZO and (f) Li_2CO_3:MZO ETL. Inset figures exhibit temperature images of each EODs captured by IR camera during the constant current stress. Here, the constant current stress condition at each EODs was fixed as 50 mA for 30 min in 4 mm^2 active area.

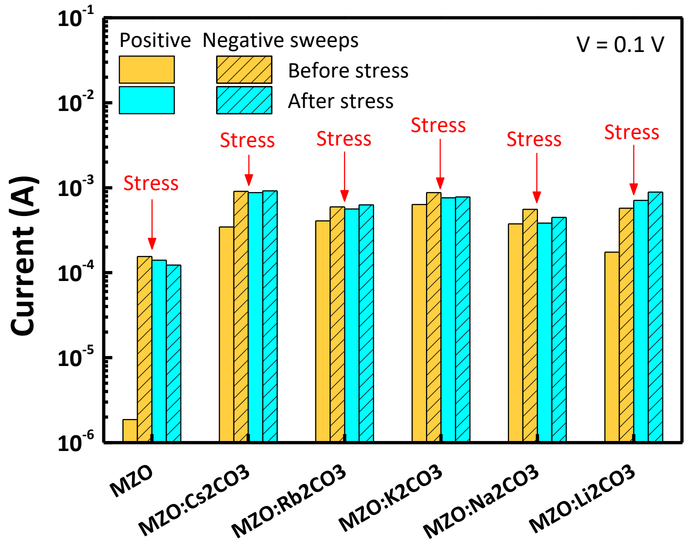

Figure S3. Summarized electrical stability (hysteresis) characteristics of EODs with alkali metal carbonate blended MZO ETLs at 0.1 V before and after current stress (50 mA for 30 min).

Table S2. Summarized lifetime and current efficiency characteristics of inverted R-QLEDs reported in literatures.

Year	Structure	CE[1)] (cd/A)	EL_{peak} (nm)	L_{int} (cd/m^2)	Lifetime (@ T_{95}, hr)	Ref.
**2013**	ITO/AZO:Cs_2CO_3/QDs/TCTA/NPB/HAT-CN/Al	~4.5	630	N/A	N/A	[1]
**2015**	ITO/ZnO NPs/Cs_2CO_3/QDs/NPB/LG101/Al	~19	620	2000	~20	[2]
**2018**	ITO/ZnO:Cs_2CO_3/QDs/p-TPD/PEDOT:PSS:Triton X-100/Al	~ 5	622	N/A	N/A	[3]
**2018**	ITO/ZnO:CsN_3/QDs/TAPC/HAT-CN/MoO_3/Al	N/A	630	N/A	N/A	[4]
**2019**	ITO/LZO/MZO:Rb_2CO_3/QDs/TCTA/NPB/HAT-CN/Al	~13	623	1,000	~200	[5]
**This study**	ITO/LZO/MZO:Cs_2CO_3/QDs/TCTA/NPB/HAT-CN/Al	10.1	623	1,000	~407	-
	ITO/LZO/MZO:Rb_2CO_3/QDs/TCTA/NPB/HAT-CN/Al	11.7			~620	

[1)] CE values were measured at high luminance of 50,000 cd/m^2.

References

[1] H-M. Kim, A.R.M. Yusoff, J-H. Youn and J. Jang. "Inverted Quantum-dot Light Emitting Diodes with Cesium Carbonate doped Aluminium-Zinc-Oxide as the Cathode Buffer Layer for High Brightness" *J. Mater. Chem. C*, **2013**, 1, 3924-3930.

[2] Y. Dong, J-M. Caruge, Z. Zhou, C. Hamilton, Z. Popovic, J. Ho, M. Stevenson, G. Liu, V. Bulovic, M. Bawendi, P. T. Kazlas, J. Steckel and S. Coe-Sullivan. "Ultra-Bright, Highly Efficient, Low Roll-off Inverted Quantum-dot Light Emitting Devices (QLEDs)" *Dig. Tech. Pap. - Soc. Inf. Disp. Int. Symp*, **2015**, 46, 270-273.

[3] M. A. Triana, H. Chen, D. Zhang, R. J. Camargo, T. Zhai, S. Duhm and Y. Dong. "Bright Inverted Quantum-dot Light Emitting Diodes by All-solution Processing" *J. Mater. Chem. C*, **2018**, 6, 7487-7492.

[4] J. Pan, C. Wei, L. Wang, J. Zhuang, Q. Huang, W. Su, Z. Cui, A. Nathan, W. Lei and J. Chen. "Boosting the Efficiency of Inverted Quantum-dot Light-emitting Diodes by Balancing Charge Densities and Suppressing Exciton Quenching through Band Alignment" *Nanoscale*, **2018**, 10, 592-602.

[5] Y. Lee, H-M. Kim, J. Kim and J. Jang. "Remarkable Lifetime Improvement of Quantum-dot Light Emitting Diodes by Incorporating Rubidium Carbonate in Metal-oxide Electron Transport Layers" *J. Mater. Chem. C*, **2019**, 10082-10091.
